# Exosome‐Loaded Engineered circBDNF Promotes Spinal Cord Injury Repair Through the PI3K/AKT/mTOR Signaling Axis

**DOI:** 10.1002/cns.70784

**Published:** 2026-03-19

**Authors:** Gang Li, Ganggang Kong, Cheng Gu, Di Zhang, Yong Wan, Baoshu Xie

**Affiliations:** ^1^ Department of Orthopaedics, Union Hospital, Tongji Medical College Huazhong University of Science and Technology Wuhan Hubei Province China; ^2^ Department of Spinal Surgery The First Affiliated Hospital of Sun Yat‐Sen University Guangzhou Guangdong Province China; ^3^ Guangdong Province Key Laboratory of Orthopaedics and Traumatology Guangzhou Guangdong China; ^4^ Department of Joint Surgery The First Affiliated Hospital of Sun Yat‐Sen University Guangzhou Guangdong Province People's Republic of China; ^5^ Department of Neurosurgery The First Affiliated Hospital of Sun Yat‐Sen University Guangzhou Guangdong Province People's Republic of China

**Keywords:** circBDNF, exosomes, neuroprotection, PI3K/AKT/mTOR, spinal cord injury

## Abstract

**Background:**

The brain‐derived neurotrophic factor (BDNF) is a potent neuroprotective factor; however, its large molecular size limits its ability to cross structural barriers such as the blood–spinal cord barrier. This study explores the therapeutic potential of exosome‐mediated delivery of engineered circular BDNF (circBDNF) to promote spinal cord injury (SCI) repair through activation of the PI3K/AKT/mTOR signaling pathway.

**Methods:**

A synthetic circBDNF sequence encoding BDNF was used to construct a circBDNF overexpression plasmid, which was transfected into HEK293T cells to generate circBDNF‐loaded exosomes (circBDNF‐EXO). These exosomes were characterized via transmission electron microscopy, nanoparticle tracking analysis, and Western blotting. In vitro, the protective effects of circBDNF‐EXO were evaluated in an oxygen–glucose deprivation/reperfusion (OGD) injury model in HT22 cells, focusing on cell viability, reactive oxygen species (ROS) levels, apoptosis, inflammation, and signaling pathways. In vivo, a T10 SCI mouse model was employed to assess therapeutic efficacy, using behavioral, electrophysiological, histological, and molecular analyses.

**Results:**

In vitro, circBDNF‐EXO treatment significantly increased BDNF expression, enhanced cell viability, reduced ROS levels, mitigated inflammation, and inhibited apoptosis in HT22 cells following OGD injury. In vivo, administration of circBDNF‐EXO resulted in improved motor function recovery, evidenced by increased Basso Mouse Scale scores, enhanced gait coordination, and better motor‐evoked potentials. Histological analyses demonstrated elevated BDNF expression, decreased apoptosis, reduced oxidative stress, and enhanced axonal regeneration in the injured spinal cord. Mechanistically, circBDNF‐EXO activated TrkB receptors and upregulated the PI3K/AKT/mTOR signaling pathway, as confirmed by Western blot analysis.

**Conclusion:**

Exosome‐mediated delivery of circBDNF promotes SCI repair by activating the PI3K/AKT/mTOR pathway, suppressing apoptosis, oxidative stress, and inflammation, and enhancing axonal regeneration. This innovative approach holds substantial promise for SCI treatment and deserves further exploration in preclinical and clinical studies.

## Graphical abstract

Engineered exosomes carrying circBDNF promote spinal cord injury repair by activating the PI3K/AKT/mTOR pathway. This treatment reduces apoptosis and inflammation, enhances axonal regeneration, and restores motor function, offering a promising neurotrophic strategy for spinal cord repair.

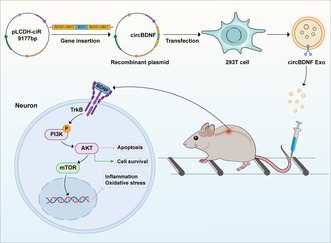

## 1 Introduction

Spinal cord injury (SCI) is a devastating condition that results from damage to the spinal cord, often leading to severe and permanent neurological deficits. Despite advances in medical research, effective treatments for SCI remain limited [1]. Brain‐derived neurotrophic factor (BDNF) is a 274‐amino‐acid protein belonging to the neurotrophin family and plays a critical role in the repair and regeneration of the nervous system. In the context of SCI, BDNF promotes neuronal survival and axonal regeneration, which are essential for functional recovery [2].

Circular RNAs (circRNAs) are a novel class of non‐coding RNAs characterized by their covalently closed loop structure, which distinguishes them from linear RNAs. They are generated through a process called back‐splicing, where the 3′ and 5′ ends of a pre‐mRNA are joined together. circRNAs are abundant, stable, and evolutionarily conserved across different species. Recent studies have revealed that circRNAs play crucial roles in various biological processes, including gene expression regulation, microRNA (miRNA) sponging, and interaction with RNA‐binding proteins [3]. CircRNAs have attracted significant interest as potential therapeutic targets and biomarkers for various diseases, including neurological disorders such as SCI [4, 5].

Compared to linear mRNA, circRNA possesses a high degree of stability due to its covalently closed loop structure, which safeguards it against degradation by exonucleases. While only a limited number of endogenous circRNAs have been identified as capable of serving as templates for protein translation, it is possible to engineer circRNAs for this purpose. Despite lacking the necessary components for cap‐dependent translation, circRNAs can be modified to facilitate protein synthesis by incorporating an internal ribosome entry site (IRES) or adding the m6A modification just upstream of the open reading frame (ORF) [6]. This adaptability suggests that circRNAs could be effectively utilized as a platform for gene therapy.

Exosomes are naturally occurring lipid bilayer vesicles with a diameter of approximately 40–100 nm. They can be produced by most cells and contain a variety of molecular cargo, including miRNAs, messenger RNAs (mRNAs), circRNAs, DNA fragments, and proteins [7]. Exosomes can transfer these molecules from donor cells to recipient cells, where they exert their functions at new sites. They play crucial roles in both physiological and pathological processes, influencing signaling pathways, cell‐to‐cell communication, tumor progression, and molecular transfer [8]. Exosomes can encapsulate circRNAs to facilitate intercellular communication, offering several advantages as delivery vehicles. They provide a protective environment that prevents circRNAs from degrading in the bloodstream. Additionally, exosomes possess a natural ability to cross the blood–spinal cord barrier, enabling them to efficiently reach the site of SCI. These characteristics significantly enhance the therapeutic potential of circRNA‐based treatments [9, 10].

In this study, we firstly constructed a circBDNF with the ability to translate into BDNF within cells and encapsulated it in exosomes for targeted delivery to the SCI area, thereby achieving the neuroprotective effects of BDNF.

## 2 Materials and Methods

### 2.1 Plasmid Construction

The target sequences, including BDNF‐ORF2, IRES, and BDNF‐ORF1, were fully synthesized and cloned into the pLCDH‐cir vector by GeneRay Biotech (Shanghai, China).

### 2.2 Cell Culture and Transfection

HEK293T cells and HT22 cells (a murine hippocampal neuronal cell line) were cultured under similar conditions. The cells were maintained in Dulbecco's Modified Eagle Medium (DMEM) supplemented with 10% fetal bovine serum (FBS) and 1% penicillin–streptomycin. They were incubated at 37°C in a humidified atmosphere with 5% CO_2_ to ensure optimal growth and viability for subsequent experiments. The cultured cells were transfected with the circBDNF pLCDH‐cir vector using Lipofectamine 3000, following the manufacturer's protocol. A control group transfected with an empty vector (CircVec) was included for comparative analysis.

### 2.3 Exosome Isolation

Exosomes were isolated from the culture media of HEK293T cells transfected with either circBDNF or circVec plasmids. After 48 h of transfection, the culture media were collected and subjected to a multi‐step ultracentrifugation process to purify exosomes. The collected culture media were first centrifuged at 300 × g for 10 min to remove cells, followed by centrifugation at 2000 × g for 20 min to eliminate cell debris. The supernatant was then subjected to ultracentrifugation at 10,000 × g for 30 min to remove larger vesicles. The supernatant was further centrifuged at 100,000 × g for 70 min using an ultracentrifuge. The pellet, containing exosomes, was washed with phosphate‐buffered saline (PBS) and centrifuged again at 100,000 × g for another 70 min. The final exosome pellet was resuspended in PBS and stored at −80°C for further experiments. The exosomes derived from circBDNF pLCDH‐cir vector‐transfected cells were labeled as circBDNF‐EXO, and those from circVec‐transfected cells as circVec‐EXO.

The exosome morphology was examined using transmission electron microscopy (TEM). Exosome size distribution and concentration were measured using nanoparticle tracking analysis (NTA, Flow NanoAnalyzer, Fuliu Biological, Xiamen, China). Exosome protein markers were confirmed by Western blot analysis using specific antibodies against CD63, TSG101, and Calnexin.

### 2.4 Oxygen–Glucose Deprivation/Reperfusion (OGD) Injury Model

To simulate OGD injury, the culture medium was replaced with glucose‐free DMEM and the cells were transferred to a hypoxia chamber (1% O_2_) for 6 h. After OGD, glucose was reintroduced by replacing the medium with normal DMEM and the cells were returned to normoxic conditions for 24 h to induce reperfusion.

### 2.5 Cell Viability Assay

A cell viability assay CCK‐8 was performed 24 h after reperfusion to assess the protective effect of circBDNF against OGD‐induced injury. Absorbance was measured using a microplate reader to quantify the viable cells.

### 2.6 Apoptosis Analysis

For apoptosis analysis, HT22 cells transfected with circBDNF were subjected to TUNEL staining to evaluate apoptotic cell levels. This was complemented by Western blotting to assess the expression of key apoptotic markers, including Cleaved caspase‐3, Bax, and Bcl‐2.

### 2.7 Quantitative Real‐Time Polymerase Chain Reaction (qRT‐PCR)

Total RNA was isolated from cells and tissues using Trizol reagent (Life Tech, Carlsbad, CA) according to the manufacturer's protocol. RNA purity and concentration were determined using a NanoDrop 2000 spectrophotometer (Thermo Fisher Scientific). Complementary DNA (cDNA) was synthesized from 1 μg of total RNA using the High‐Capacity RNA‐to‐cDNA Kit (Life Tech). Quantitative real‐time PCR was carried out on a LightCycler 480 system (Roche, Basel, Switzerland) with SYBR Green Master Mix (Roche). Each sample was analyzed in triplicate, and relative gene expression levels were calculated using the 2‐ΔΔCt method, normalized to GAPDH. Primer sequences used for the analysis are provided in Table S1.

### 2.8 Spinal Cord Injury Model and circBDNF Treatment

Male C57BL/6 mice (8 weeks old) were used in this study. All animal procedures were conducted in accordance with institutional guidelines for animal care and use. A T10 spinal cord clamp injury model was employed to induce SCI. The mice were anesthetized with isoflurane, and a laminectomy was performed at the T10 vertebral level to expose the spinal cord. A modified aneurysm clip (force of 35 g) was applied for 1 min to create a consistent and reproducible SCI. Exosomes encapsulating circBDNF were prepared and purified from transfected cells. Immediately after SCI, the mice received an intrathecal injection of exosome‐derived circBDNF (10 μL) at the injury site. The injections were repeated at days 1 and 3 post‐injury to ensure sustained delivery of circBDNF.

### 2.9 Behavioral Assessments

Basso Mouse Scale (BMS): The recovery of motor function was assessed using the BMS from 1 week to 16 weeks post‐injury. The BMS scores range from 0 (complete paralysis) to 9 (normal locomotion) and allow for the evaluation of hindlimb movement and coordination.

Gait Analysis: A digital gait analysis system was used to assess the walking patterns of the mice. Parameters such as stride length, step width, and paw placement were recorded to evaluate the quality of locomotion and coordination.

### 2.10 Immunofluorescence Staining

The sections of mouse spinal cord tissues were then blocked with 10% BSA and incubated overnight at 4°C with primary antibodies, such as anti‐GFAP, anti‐Iba1, anti‐NeuN, anti‐GAP43, and other relevant markers. The following day, tissue sections were incubated with secondary antibodies. Nuclei were counterstained with 4′,6‐diamidino‐2‐phenylindole (DAPI). Immunofluorescence images were captured using a confocal microscope. Fluorescence quantification was performed by measuring the intensity of the fluorescent signal and the percentage of positive staining in three distinct regions of interest (ROI) within each sample. Antibodies used are listed in Table S2.

### 2.11 Western Blot Analysis

Protein samples from cells or tissues were prepared, and an equal amount of protein was loaded onto SDS‐PAGE gels with appropriate concentrations based on the molecular weight of the target proteins. Following electrophoretic separation, proteins were transferred onto PVDF membranes (Millipore, Billerica, MA, USA). The membranes were then blocked with blocking buffer (Epizyme, Shanghai, China) for 1 h at room temperature. Subsequently, the membranes were incubated overnight at 4°C with primary antibodies targeting the proteins of interest: Bcl‐2, Cleaved caspase‐3, Cleaved caspase‐9, BDNF, TrkB, p‐PI3K, p‐AKT, and p‐mTOR. The following day, membranes were incubated with HRP‐conjugated secondary antibodies for 2 h at room temperature. Protein bands were visualized using ECL reagent and imaged with the Chemiluminescent Imaging System (Tanon 5200, China). The intensity of protein bands was semi‐quantitatively analyzed using ImageJ software. Each experiment was performed in triplicate to ensure reproducibility. Antibodies used are listed in Table S3.

### 2.12 Statistical Analysis

Statistical analysis was performed using SPSS software, version 22 (IBM, Armonk, NY, USA). Data are presented as the mean ± standard deviation (SD). For comparisons between two groups, Student's *t*‐tests were conducted, while differences among multiple groups were analyzed using one‐way analysis of variance (ANOVA) followed by Tukey's post hoc test. For repeated measurements over time, repeated‐measures ANOVA was applied. Nonparametric data were analyzed using the Mann–Whitney U test or the Kruskal–Wallis test, as appropriate. Statistical significance was defined as *p* < 0.05.

## 3 Results

### 3.1 Constructing a circBDNF Plasmid Capable of Translating BDNF

**FIGURE 1:**
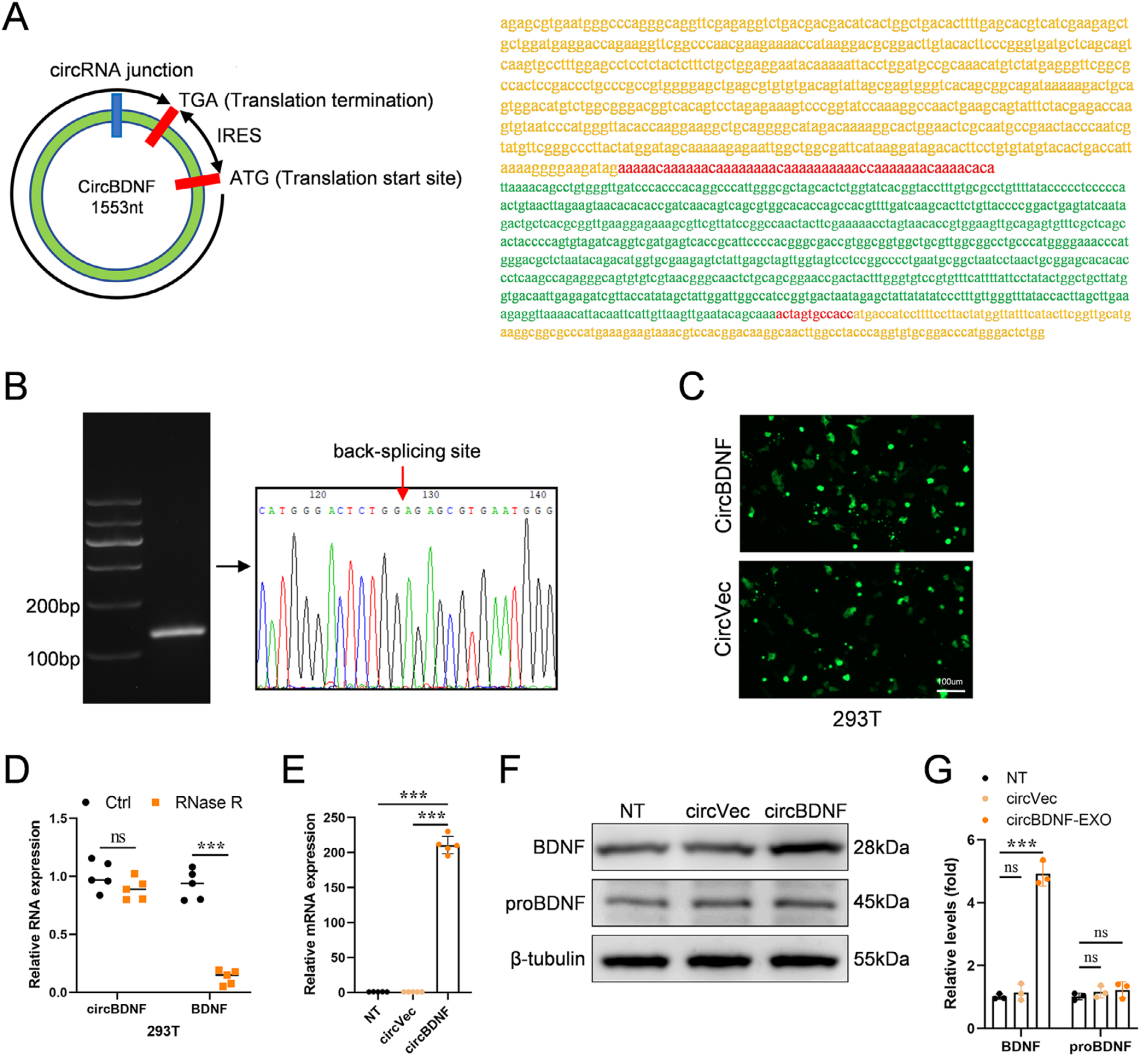
Validation and characterization of circBDNF. (A) Schematic representation of the circBDNF structure, including the back‐splicing junction, IRES, and translation start sites (ATG and TGA). The sequence of circBDNF is displayed on the right. (B) Agarose gel electrophoresis showing the specific amplification of circBDNF using divergent primers and Sanger sequencing confirming the back‐splicing junction (red arrow). (C) Fluorescence microscopy analysis of GFP expression in cells transfected with circBDNF or circVec plasmids. (D) RNase R resistance assay demonstrating the stability of circBDNF compared to linear BDNF mRNA. (E) Quantitative RT‐PCR analysis showing increased expression of circBDNF in cells transfected with circBDNF plasmids compared to the non‐transfected (NT) and circVec groups. (F and G) Western blot analysis and corresponding quantification illustrating enhanced mature BDNF protein expression in the circBDNF‐transfected group compared to the NT and circVec groups. Data are presented as mean ± SD. ***p < 0.001; ns, not significant.

The BDNF‐ORF2, IRES, and BDNF‐ORF1 sequences were entirely synthesized and inserted into the pLCDH‐cir plasmid vector, which served as the template for in vitro transcription of circBDNF (Figure 1A).

To validate the successful expression and circularization of circBDNF, we transfected the constructed pLCDH‐cir plasmid vector into cultured HEK293T cells. GFP protein expression was validated with a fluorescence microscope, demonstrating successful transfection and GFP‐tagged plasmid expression. The circular structure of circBDNF was confirmed through several approaches. RT‐PCR amplified the back‐splicing junction unique to circRNAs, while RNase H‐mediated specific cleavage further distinguished the circular form from linear transcripts. Sanger sequencing definitively verified the presence of the back‐splicing junction, providing conclusive evidence of RNA circularization (Figure 1B–E).

Western blot analysis showed abundant BDNF protein expression in HEK293T cells transfected with the circBDNF plasmid, demonstrating that circBDNF effectively translated BDNF protein in vitro (Figure 1F,G). These findings collectively establish the successful construction of circBDNF, its circular structure, and its functional capacity to produce BDNF protein.

### 3.2 Exosome Isolation and Characterization

**FIGURE 2:**
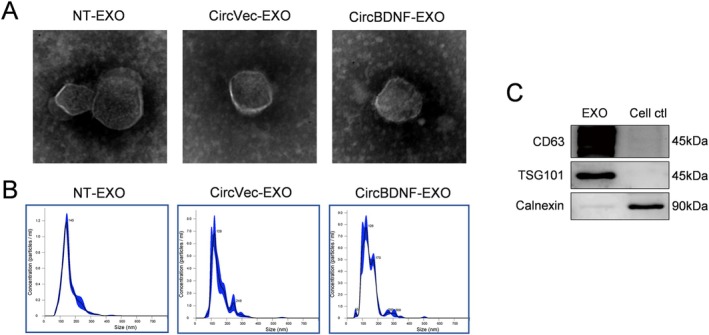
Characterization of exosomes derived from circBDNF‐transfected cells. (A) TEM images showing the morphology of exosomes derived from NT‐EXO, CircVec‐EXO, and CircBDNF‐EXO groups. (B) NTA demonstrating the size distribution of exosomes. (C) Western blot analysis of exosome markers CD63 and TSG101, confirming the identity of exosomes. The absence of calnexin, a negative marker, indicates no cellular contamination.

Exosomes were isolated from the culture media of cells transfected with either NT, circBDNF, or circVec using ultracentrifugation, a standard method for exosome purification. The isolated exosomes were designated as NT‐EXO, circBDNF‐EXO, and circVec‐EXO, respectively. TEM imaging revealed the characteristic cup‐shaped morphology of exosomes, with diameters primarily ranging from 120 to 150 nm, confirming their identity and structural integrity (Figure 2A). NTA further validated these findings, showing that the majority of exosome particles across all groups were within the 120–150 nm size range, with similar distribution profiles and concentrations (Figure 2B). These results demonstrate the successful isolation and comparable physical properties of exosomes derived from different transfection conditions. We further performed Western blot analysis for key exosomal markers. Specifically, we detected the presence of at least two positive markers—CD63 and TSG101—indicating the purity and identity of the exosomes. Additionally, we assessed the absence of the endoplasmic reticulum protein Calnexin, which served as a negative control to confirm that the preparation was free from cellular contamination (Figure 2B). These characterization methods collectively confirmed the successful extraction and proper identification of NT‐EXO, circBDNF‐EXO, and circVec‐EXO, ensuring their suitability for subsequent functional assays.

### 3.3 CircBDNF‐EXO Protect HT22 Cells From OGD Injury

**FIGURE 3:**
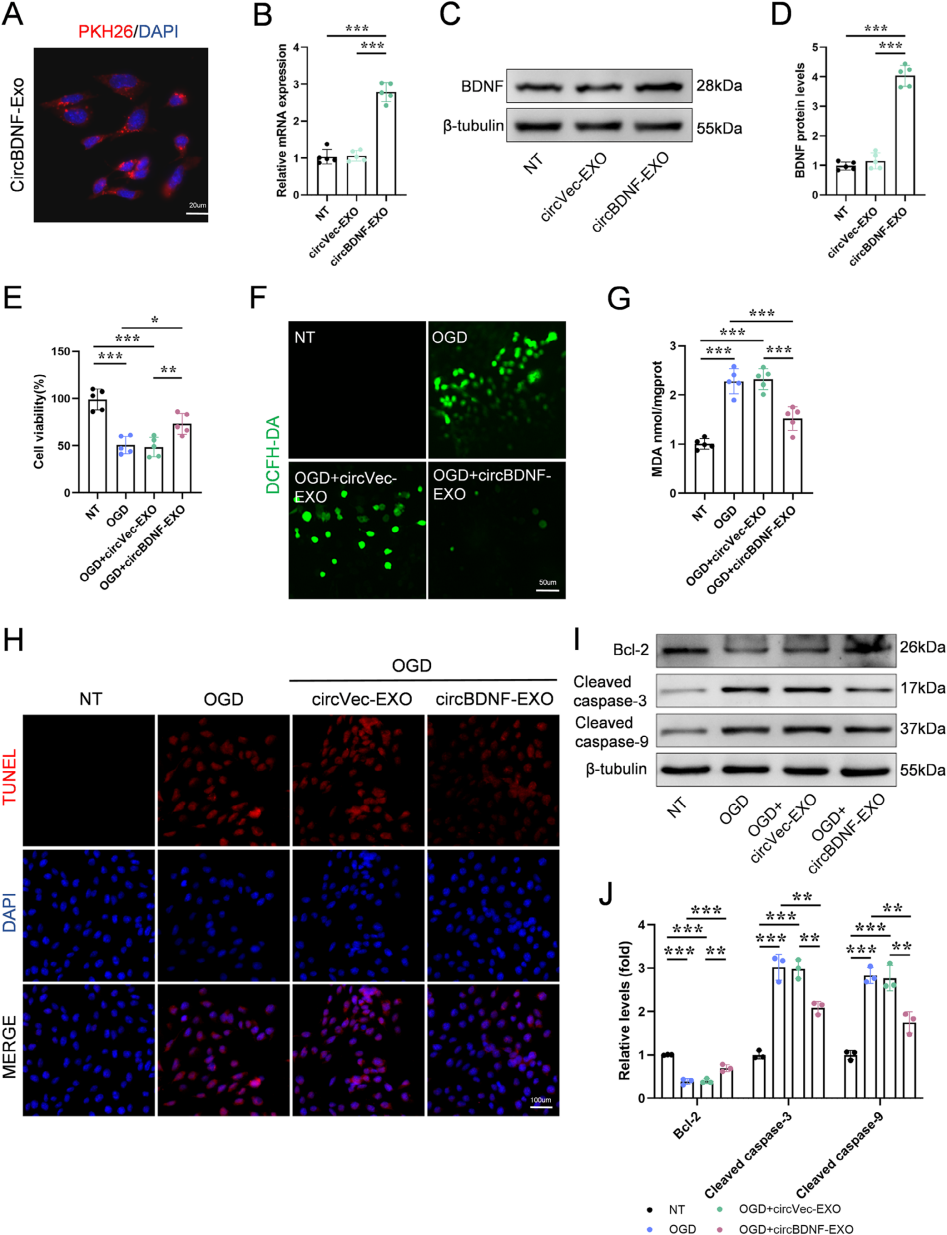
CircBDNF‐EXO protects neuronal cells from oxidative stress and apoptosis under OGD conditions. (A) Uptake of PKH26‐labeled circBDNF‐EXO by neuronal cells, as shown by red fluorescence colocalized with DAPI nuclear staining. (B) Quantitative RT‐PCR analysis of BDNF mRNA expression in neuronal cells treated with NT, circVec‐EXO, or circBDNF‐EXO under OGD conditions. CircBDNF‐EXO significantly upregulated BDNF mRNA levels. (C and D) Western blot and quantitative analysis of BDNF protein expression. CircBDNF‐EXO treatment markedly increased BDNF protein levels compared to NT and circVec‐EXO groups. (E) Cell viability assay showing that circBDNF‐EXO treatment significantly improved neuronal cell viability under OGD conditions compared to NT and circVec‐EXO groups. (F) Detection of intracellular ROS using DCFH‐DA staining. CircBDNF‐EXO treatment significantly reduced ROS levels under OGD conditions. (G) MDA assay showing reduced lipid peroxidation in cells treated with circBDNF‐EXO under OGD conditions. (H) TUNEL staining of neuronal cells under OGD conditions, showing a reduction in apoptotic cells following circBDNF‐EXO treatment compared to NT and circVec‐EXO groups. (I and J) Western blot and quantitative analysis of apoptosis‐related proteins Bcl‐2, Cleaved caspase‐3, and Cleaved caspase‐9. CircBDNF‐EXO treatment decreased cleaved caspase‐3 and cleaved caspase‐9 levels and increased Bcl‐2 expression, indicating reduced apoptosis. *p < 0.05; **p < 0.01; ***p < 0.001.

To simulate the ischemic and hypoxic conditions associated with SCI in vivo, we subjected HT22 cells to OGD injury. CircBDNF‐EXO were labeled with PKH26 and co‐cultured with HT22 cells. Fluorescence microscopy confirmed the successful uptake of circBDNF‐EXO by the cells, indicating efficient delivery of circBDNF into HT22 cells (Figure 3A).

Quantitative RT‐PCR analysis revealed that neuronal cells treated with circBDNF‐EXO under OGD conditions exhibited a significant upregulation of BDNF mRNA levels compared to cells treated with NT or circVec‐EXO (Figure 3B). This result demonstrates that circBDNF‐EXO effectively enhanced BDNF transcription in the target cells. Western blot analysis revealed a significant increase in BDNF protein levels in the circBDNF‐EXO treated group compared to the circVec‐EXO and NT groups, confirming that circBDNF was effectively translated into functional BDNF protein (Figure 3C,D). This upregulation of BDNF expression suggests that circBDNF‐EXO treatment successfully activated neuroprotective pathways.

Cell viability was assessed using the CCK8 assay, which showed that circBDNF‐EXO treatment significantly enhanced HT22 cell survival following OGD injury. These findings indicate that circBDNF confers protection against OGD‐induced damage (Figure 3E).

To further explore the protective mechanisms, we assessed reactive oxygen species (ROS) levels using the DCFH‐DA fluorescent probe. CircBDNF‐EXO treatment significantly reduced ROS levels, demonstrating its antioxidative effects (Figure 3F). Lipid peroxidation, measured by malondialdehyde (MDA) levels, was also markedly decreased in the circBDNF‐EXO treated group, further supporting its role in mitigating oxidative stress (Figure 3G).

Apoptosis was evaluated using TUNEL staining, which showed a reduced rate of apoptosis in HT22 cells treated with circBDNF‐EXO compared to controls (Figure 3H). This anti‐apoptotic effect was corroborated by Western blot analysis, which revealed that circBDNF‐EXO treatment upregulated the expression of the anti‐apoptotic protein BCL‐2 while downregulating the pro‐apoptotic proteins cleaved caspase‐3 and cleaved caspase‐9 (Figure 3I,J).

In summary, circBDNF‐EXO provide robust protection against OGD‐induced injury in HT22 cells by promoting cell survival, reducing oxidative stress, and inhibiting apoptosis. These results highlight circBDNF‐EXO as a promising therapeutic strategy for treating conditions such as SCI.

### 3.4 CircBDNF‐EXO Alleviates Inflammation, Oxidative Stress, and Apoptosis While Enhancing Neuronal Survival After SCI

**FIGURE 4:**
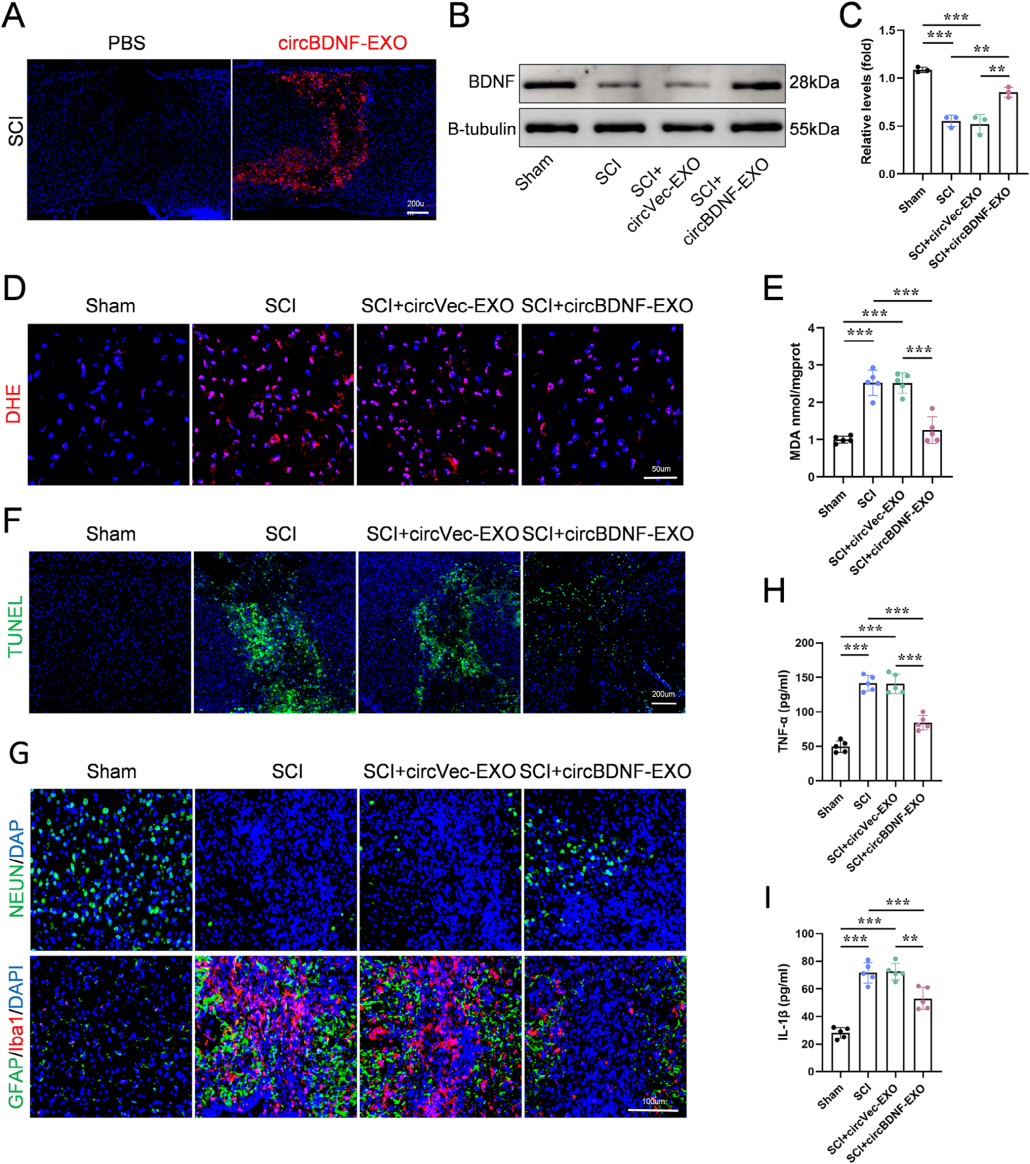
CircBDNF‐EXO reduces inflammation, oxidative stress, and apoptosis while promoting neuronal survival after SCI. (A) Distribution of circBDNF‐EXO in the injured spinal cord, visualized by fluorescence microscopy. (B and C) Western blot analysis and quantification of BDNF protein expression in spinal cord tissues across different groups. (D) Representative DHE staining images showing ROS levels in spinal cord tissues. (E) Quantification of MDA levels, a marker of oxidative stress, in spinal cord tissues across groups. (F) TUNEL staining showing apoptosis in spinal cord tissues across different treatment groups. (G) Representative immunofluorescence images of NeuN, GFAP, and Iba1 staining to assess neuronal survival, glial activation, and inflammation. (H and I) Quantification of pro‐inflammatory cytokines TNF‐α and IL‐1β levels in spinal cord tissues across groups. Data are presented as mean ± SD. *p < 0.05, **p < 0.01, ***p < 0.001. Scale bars are indicated in the images.

To evaluate the therapeutic effects of circBDNF‐EXO in SCI, histological analyses at 3 days post‐injury demonstrated that circBDNF‐EXO significantly accumulated in the injured spinal cord tissue, as evidenced by strong red fluorescence in the exosome‐treated regions compared to the PBS control (Figure 4A). This was accompanied by a marked upregulation of BDNF protein levels, as shown by Western blot and quantitative analysis, confirming the successful delivery and functional expression of circBDNF in the injured tissue (Figure 4B,C).

CircBDNF‐EXO treatment effectively reduced oxidative stress in the injured spinal cord at 3 days post‐injury. DHE staining revealed a notable reduction in ROS production in the circBDNF‐EXO group compared to SCI and SCI + circVec‐EXO groups (Figure 4D). Consistently, biochemical measurements showed significantly decreased malondialdehyde (MDA) levels in the circBDNF‐EXO‐treated group, indicating lower lipid peroxidation (Figure 4E).

Apoptosis was significantly attenuated in the circBDNF‐EXO group 3 days after SCI. TUNEL staining showed a reduced number of apoptotic cells in circBDNF‐EXO‐treated spinal cord tissues compared to controls (Figure 4F).

In addition to its anti‐apoptotic and antioxidative effects, circBDNF‐EXO treatment promoted neuronal survival and reduced inflammation at the injury site 3 days post‐injury. Immunofluorescence staining revealed an increased number of NeuN‐positive neurons in the circBDNF‐EXO group, highlighting its neuroprotective effects (Figure 4G). Moreover, markers of inflammation, including GFAP and Iba1, were markedly decreased in the circBDNF‐EXO‐treated group compared to controls, demonstrating reduced astrocytic and microglial activation (Figure 4G). This anti‐inflammatory effect was further supported by significantly lower levels of pro‐inflammatory cytokines TNF‐α and IL‐1β in the circBDNF‐EXO group (Figure 4H,I).

These findings collectively demonstrate that circBDNF‐EXO reduces inflammation, mitigates oxidative stress and apoptosis, and enhances neuronal survival 3 days after SCI, underscoring its therapeutic potential for SCI.

### 3.5 CircBDNF‐EXO Activates the PI3K/AKT/mTOR Pathway and Modulates Apoptotic Proteins After SCI

**FIGURE 5:**
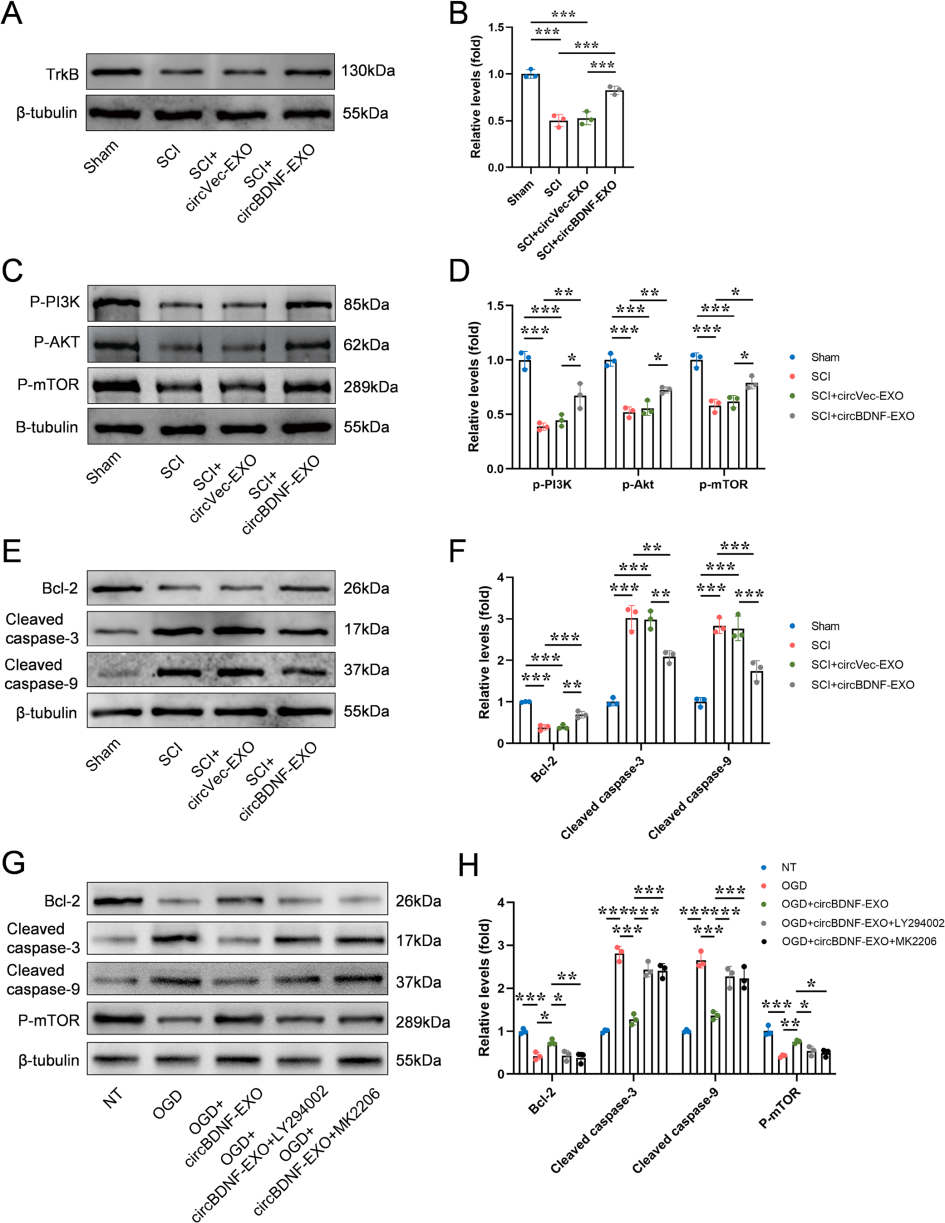
CircBDNF‐EXO treatment enhances PI3K/AKT/mTOR signaling after SCI. (A and B) Western blot analysis and corresponding quantification of TrkB expression in spinal cord tissues. (C and D) Western blot analysis and quantification of p‐PI3K, p‐AKT, and p‐mTOR. (E and F) Western blot analysis and quantification of apoptosis‐related proteins. (G and H) Western blot analysis and quantification of apoptosis‐related proteins and p‐mTOR under circBDNF‐EXO treatment with or without PI3K or AKT inhibitors (LY294002 and MK2206). Data are expressed as mean ± SD. *p < 0.05, **p < 0.01, ***p < 0.001.

Mechanistic studies at 3 days post‐SCI revealed that circBDNF‐EXO treatment significantly upregulated TrkB protein expression compared to the SCI and SCI + circVec‐EXO groups, as shown by Western blot analysis (Figure 5A,B). The increase in TrkB expression suggests that circBDNF‐EXO effectively activates the TrkB receptor, a key mediator of BDNF signaling.

Further downstream, the activation of the PI3K/AKT/mTOR pathway was confirmed by the significant increase in phosphorylation levels of PI3K, AKT, and mTOR in the circBDNF‐EXO‐treated group compared to the SCI and SCI + circVec‐EXO groups (Figure 5C,D). These findings demonstrate that circBDNF‐EXO activates the PI3K/AKT/mTOR pathway, which is essential for promoting cell survival and reducing apoptosis.

In addition to pathway activation, circBDNF‐EXO treatment significantly modulated the expression of apoptosis‐related proteins. The expression of the anti‐apoptotic protein Bcl‐2 was markedly upregulated, while the pro‐apoptotic proteins cleaved caspase‐3 and cleaved caspase‐9 were significantly downregulated in the circBDNF‐EXO group compared to controls (Figure 5E,F). However, these anti‐apoptotic effects were largely abolished when the cells were pretreated with the PI3K inhibitor LY294002 or the Akt inhibitor MK2206 (Figure 5G,H). These changes indicate a robust inhibition of the apoptotic cascade, further supporting the neuroprotective role of circBDNF‐EXO.

These results suggest that circBDNF‐EXO exerts its therapeutic effects in SCI by activating the PI3K/AKT/mTOR signaling pathway and modulating the expression of key apoptotic proteins to promote cell survival and functional recovery.

### 3.6 Therapeutic Efficacy of circBDNF‐EXO in Enhancing Motor Recovery and Neural Regeneration After SCI

**FIGURE 6:**
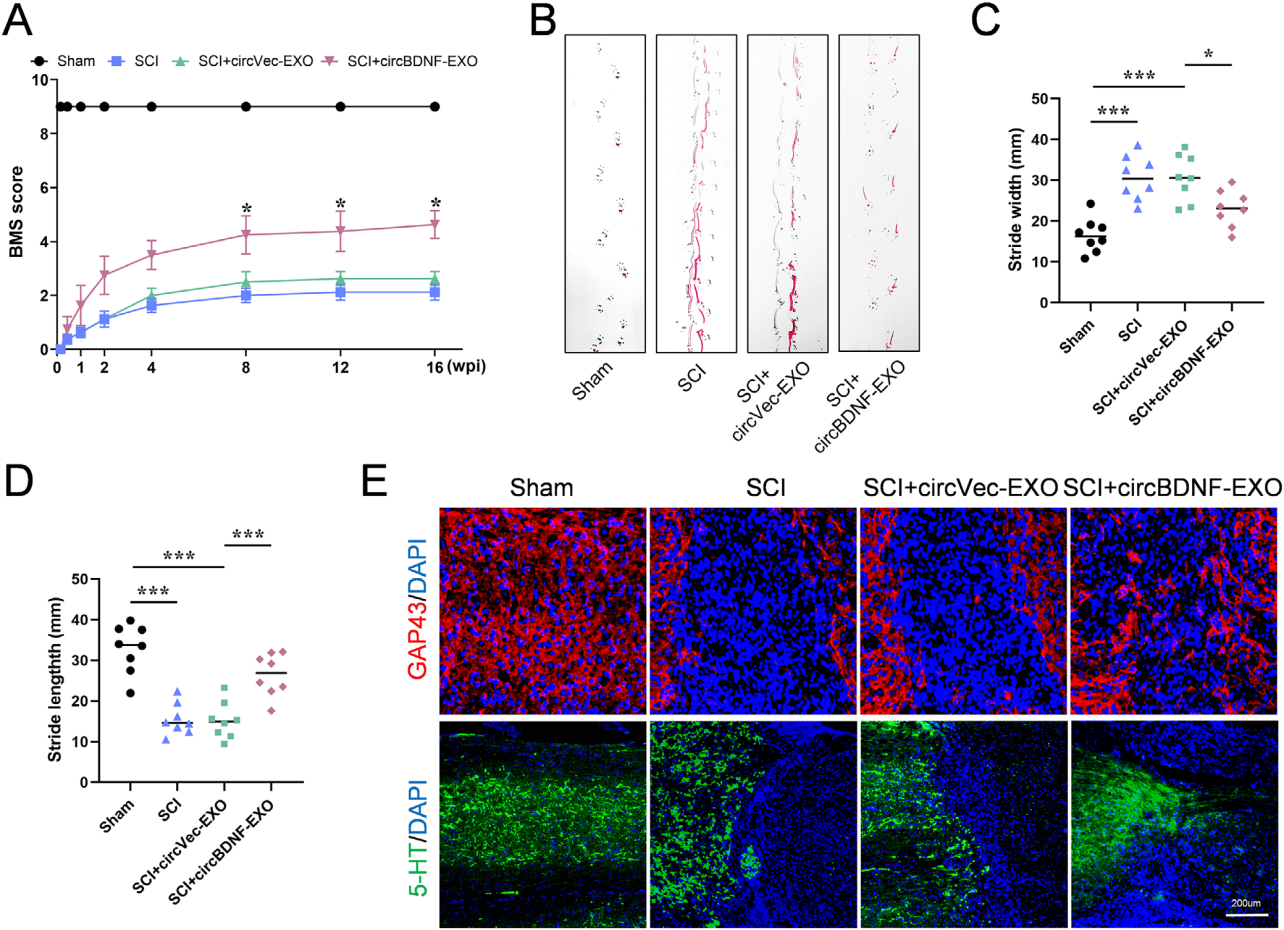
circBDNF‐EXO promotes functional recovery and axonal regeneration following SCI. (A) BMS scores evaluating locomotor recovery in Sham, SCI, SCI + circVec‐EXO, and SCI + circBDNF‐EXO groups at various time points post‐injury (*n* = 8 per group). (B, C, and D) Gait analysis reveals improvements in stride width and length in SCI + circBDNF‐EXO‐treated mice compared to controls. (E) Immunofluorescence staining for GAP43 and 5‐HT in spinal cord sections across groups, indicating axonal regeneration. *p < 0.05, **p < 0.01, ***p < 0.001.

Behavioral assessments demonstrated that circBDNF‐EXO treatment significantly improved motor function recovery. Mice receiving circBDNF‐EXO exhibited markedly higher BMS scores compared to the SCI and SCI + circVec‐EXO groups. These functional improvements were evident as early as eight weeks post‐injury and persisted through 16 weeks, highlighting sustained recovery (Figure 6A). Furthermore, gait analysis confirmed the therapeutic benefits, as circBDNF‐EXO‐treated mice displayed more consistent and coordinated step patterns, with significantly enhanced stride length and width compared to controls (Figure 6B–D).

Histological analyses revealed that circBDNF‐EXO treatment promoted the regeneration of axonal growth eight weeks post‐SCI. Immunostaining of spinal cord tissues showed increased expression of GAP‐43 and 5‐HT, both critical markers of axonal growth and synaptic remodeling, in the circBDNF‐EXO‐treated group compared to controls (Figure 6E). These findings suggest that circBDNF‐EXO facilitates axonal regeneration and the restoration of neural networks, providing a structural basis for the observed functional recovery.

## 4 Discussion

This study demonstrates that exosome‐loaded engineered circBDNF significantly promotes neurological recovery following SCI, primarily through the enhanced expression of BDNF and activation of the PI3K/AKT/mTOR signaling pathway. These findings contribute to the growing body of literature highlighting the therapeutic potential of circRNAs in neurological injuries.

BDNF is a critical neurotrophic factor; its significant role in promoting neural repair following SCI has been well established. However, due to the large molecular size of BDNF, it is challenging to effectively cross the blood–spinal cord barrier (BSCB) [11]. Traditional methods, such as direct spinal injection of BDNF or viral vectors carrying the BDNF gene, pose risks of spinal tissue damage and are not feasible for routine clinical application. Thus, developing non‐invasive strategies to enhance BDNF delivery across the BSCB and increase its bioavailability in the central nervous system has become an urgent therapeutic need for SCI.

Traditional brain and spinal cord‐targeting delivery systems have significant limitations. Inorganic nanoparticles pose unresolved toxicity issues, micelles are structurally limited in delivering water‐soluble drugs, and liposomes suffer from poor stability, limited availability of surface groups, and steric hindrance that makes it difficult to conjugate ligands to their surfaces [12, 13, 14]. In contrast, exosomes, as naturally occurring extracellular vesicles, offer significant advantages as drug delivery vehicles. These include their ability to cross biological barriers like the BSCB, low immunogenicity, inherent stability, and efficient uptake by recipient cells [15, 16]. Additionally, exosomes can be engineered to carry therapeutic molecules such as circRNAs, making them an ideal candidate for targeted CNS delivery [17]. Recent advances in exosome‐based delivery systems have demonstrated their potential in neural repair. For instance, Yang et al. showed that exosome‐mediated delivery of miR‐124 promoted cortical neurogenesis and provided protection against ischemic brain injury by enhancing the identity of cortical neural progenitors [18]. Similarly, studies on circ‐WDfy3 encapsulated in exosomes demonstrated its ability to promote spinal cord repair by regulating ferroptosis through the miR‐138‐5p/GPX4 pathway in SCI models [19]. Building on this growing body of evidence, our study is the first to develop and utilize engineered exosomes loaded with circBDNF to target SCI. CircBDNF, a circular RNA with the unique ability to be translated into BDNF within cells, was encapsulated in exosomes and administered intravenously. This delivery strategy allowed circBDNF‐EXO to efficiently cross the BSCB and accumulate in the injured spinal cord region. Notably, circBDNF‐EXO treatment resulted in a significant increase in BDNF expression within local tissue cells, validating the feasibility of using exosomes as a targeted delivery system. The circular structure of circRNAs offers several advantages over linear RNAs or mRNA therapies. CircRNAs are inherently resistant to exonuclease‐mediated degradation, providing enhanced stability and enabling sustained BDNF production at the injury site. Unlike linear BDNF mRNA or recombinant protein therapies, which are limited by their short half‐life and rapid clearance, circBDNF offers a durable and localized source of neurotrophic support [20, 21].

The therapeutic efficacy of circBDNF‐EXO in SCI recovery is demonstrated through its multi‐faceted neuroprotective and regenerative mechanisms. TUNEL staining indicated that circBDNF‐EXO treatment effectively inhibited apoptosis, a finding further supported by Western blot analyses. Specifically, circBDNF‐EXO significantly upregulated the anti‐apoptotic protein Bcl‐2 while downregulating the pro‐apoptotic proteins cleaved caspase‐3 and cleaved caspase‐9. These results strongly suggest that apoptosis inhibition is a primary neuroprotective mechanism of circBDNF‐EXO. By reducing neuronal apoptosis, circBDNF‐EXO minimizes secondary damage, preserving a greater amount of functional neural tissues, which is critical for subsequent repair and recovery [22]. Beyond its ability to inhibit apoptosis, circBDNF‐EXO was also found to significantly reduce oxidative stress and mitigate inflammation in the injured spinal cord. Oxidative stress, driven by the excessive production of ROS, and a pro‐inflammatory microenvironment are hallmark pathological processes that exacerbate secondary injury after SCI [23]. CircBDNF‐EXO effectively lowered ROS levels and modulated the expression of inflammatory cytokines, thereby halting the neurodegenerative cascade. This dual role in reducing neurotoxic factors and promoting an anti‐inflammatory state creates a favorable environment for neural repair [24]. These findings emphasize the broad therapeutic potential of circBDNF‐EXO in addressing the complex pathological mechanisms of SCI.

In addition to its neuroprotective effects, circBDNF‐EXO demonstrated a robust capacity to promote neural regeneration, which is critical for functional recovery following SCI [2, 25]. Immunostaining for GAP43 and 5‐HT further confirmed enhanced axonal growth in the spinal cord, highlighting circBDNF‐EXO's ability to facilitate the re‐establishment of disrupted neural networks. Axonal regrowth is essential for restoring motor and sensory functions, and circBDNF‐EXO's effects in this regard represent a significant step toward overcoming one of the major barriers to SCI recovery.

Mechanistically, circBDNF‐EXO achieves these effects primarily through the translation of circBDNF into mature, biologically active BDNF protein. Once translated, BDNF binds to its high‐affinity receptor, TrkB, on the surface of target neurons. This interaction triggers the activation of downstream signaling cascades, particularly the PI3K/AKT/mTOR axis. Consistent with this, pharmacological inhibition of PI3K or AKT markedly suppresses circBDNF‐EXO‐induced mTOR phosphorylation and attenuates its anti‐apoptotic effects, further confirming the involvement of the PI3K/AKT/mTOR signaling cascade. In the context of SCI, activation of this pathway has been shown to reduce inflammation and oxidative stress, critical processes that exacerbate secondary injury [26]. By modulating the inflammatory response and reducing ROS levels, circBDNF‐EXO creates an environment conducive to neuronal survival and repair. Furthermore, the PI3K/AKT/mTOR signaling pathway supports neuronal survival by upregulating anti‐apoptotic proteins and downregulating pro‐apoptotic factors, as demonstrated in this study by increased Bcl‐2 expression and decreased cleaved caspase‐3 and cleaved caspase‐9 levels. This pathway also plays a critical role in promoting axonal growth [27, 28]. It promotes cytoskeletal remodeling, crucial for axonal elongation, and upregulates growth‐associated genes, aiding in the reconstruction of disrupted neural circuits. These findings underscore the pivotal role of the PI3K/AKT/mTOR axis in mediating the neuroprotective and regenerative effects of circBDNF‐EXO, offering a promising avenue for SCI treatment.

Despite the promising results, several limitations of the study should be acknowledged. The long‐term effects of circBDNF treatment and its potential side effects remain to be explored. Future research should also investigate the potential of combining circBDNF with other therapeutic strategies, such as transcranial magnetic stimulation, functional electrical stimulation, activity‐based therapy, or robotic‐assisted locomotor training, to enhance recovery outcomes. Additionally, incorporating RNA‐seq could provide more comprehensive mechanistic insights into the effects of circBDNF on gene expression.

Importantly, the clinical translational potential of circBDNF‐EXO is underscored by its unique advantages over traditional BDNF delivery strategies. For instance, Fu et al. demonstrated that intranasally administered BDNF‐enriched small extracellular vesicles (BDNF‐sEVs) promoted functional recovery in acute SCI models, highlighting the therapeutic synergy between sEVs and neurotrophic factors [29]. Compared to this approach, circBDNF‐EXO offers enhanced molecular stability due to the covalently closed structure of circRNAs, which resists exonuclease degradation and enables sustained BDNF production. Moreover, exosome‐based delivery provides a biocompatible and low‐immunogenic platform capable of crossing the blood–spinal cord barrier and achieving targeted accumulation in injured tissues. Unlike viral vector‐mediated methods such as AAV‐BDNF, which carry risks of insertional mutagenesis and long‐term immune responses, circBDNF‐EXO avoids these safety concerns, making it a more favorable candidate for clinical translation. These attributes collectively position circBDNF‐EXO as a promising and safer alternative for non‐invasive, targeted neurotrophic therapy in SCI.

In summary, our findings demonstrate that exosome‐loaded engineered circBDNF significantly promotes SCI repair by enhancing BDNF expression and activating the PI3K/AKT/mTOR signaling pathway. This treatment effectively attenuates apoptosis and inflammation, mitigates oxidative stress, and promotes axonal regeneration, leading to improved functional recovery. Collectively, these results suggest that circBDNF‐EXO not only holds great promise as a therapeutic agent for SCI but also represents a clinically translatable strategy that warrants further investigation in preclinical and clinical settings.

## Author Contributions

B.X. and Y.W. were responsible for study design and manuscript drafting. G.K. and G.L. carried out experiments. C.G. and D.Z. analyzed the data. G.K. and B.X. were responsible for manuscript editing. All authors have read and approved the final version of the manuscript.

## Funding

This research was supported by the National Natural Science Foundation of China (No. 82100124 and 82571561), the Guangzhou Science and Technology Program (202201011069), the Youth S&T Talent Support Programme of the Guangdong Provincial Association for Science and Technology (SKXRC2025563), and the Hubei ProvincialNatural Science Foundation of China (JCZRLH202601107).

## Ethics Statement

The animal experiments were approved by the Institutional Animal Care and Use Committee of Sun Yat‐sen University (Approval No: SYSU‐IACUC‐2022‐003047).

## Consent

All authors have read and approved the final version of this manuscript and consent to its publication.

## Conflicts of Interest

The authors declare no conflicts of interest.

## Supporting information


**Table S1**: Primers for qRT‐PCR analysis of gene expression.
**Table S2**: Primary antibody information for immunofluorescence
**Table S3**: Primary antibody information for Western blot.

## Data Availability

The data that support the findings of this study are available from the corresponding author upon reasonable request.

## References

[1] A. Anjum , M. D. Yazid , M. Fauzi Daud , et al., “Spinal Cord Injury: Pathophysiology, Multimolecular Interactions, and Underlying Recovery Mechanisms,” International Journal of Molecular Sciences 21, no. 20 (2020): 7533. 33066029 10.3390/ijms21207533 PMC7589539

[2] Y. Li , A. Tran , L. Graham , J. Brock , M. H. Tuszynski , and P. Lu , “BDNF Guides Neural Stem Cell‐Derived Axons to Ventral Interneurons and Motor Neurons After Spinal Cord Injury,” Experimental Neurology 359 (2023): 114259. 36309123 10.1016/j.expneurol.2022.114259

[3] W. Y. Zhou , Z. R. Cai , J. Liu , D. S. Wang , H. Q. Ju , and R. H. Xu , “Circular RNA: Metabolism, Functions and Interactions With Proteins,” Molecular Cancer 19, no. 1 (2020): 172. 33317550 10.1186/s12943-020-01286-3 PMC7734744

[4] M. M. Siddiq , C. A. Toro , N. P. Johnson , et al., “Spinal Cord Injury Regulates Circular RNA Expression in Axons,” Frontiers in Molecular Neuroscience 16 (2023): 1183315. 37692100 10.3389/fnmol.2023.1183315 PMC10483835

[5] W. Wang , C. Liu , D. He , et al., “CircRNA CDR1as Affects Functional Repair After Spinal Cord Injury and Regulates Fibrosis Through the SMAD Pathway,” Pharmacological Research 204 (2024): 107189. 38649124 10.1016/j.phrs.2024.107189

[6] R. Chen , S. K. Wang , J. A. Belk , et al., “Engineering Circular RNA for Enhanced Protein Production,” Nature Biotechnology 41, no. 2 (2023): 262–272. 10.1038/s41587-022-01393-0 PMC9931579 35851375

[7] D. Ha , N. Yang , and V. Nadithe , “Exosomes as Therapeutic Drug Carriers and Delivery Vehicles Across Biological Membranes: Current Perspectives and Future Challenges,” Acta Pharmaceutica Sinica B 6, no. 4 (2016): 287–296. 27471669 10.1016/j.apsb.2016.02.001 PMC4951582

[8] M. Colombo , G. Raposo , and C. Thery , “Biogenesis, Secretion, and Intercellular Interactions of Exosomes and Other Extracellular Vesicles,” Annual Review of Cell and Developmental Biology 30 (2014): 255–289. 10.1146/annurev-cellbio-101512-122326 25288114

[9] X. Y. Huang , Z. L. Huang , J. Huang , et al., “Exosomal circRNA‐100338 Promotes Hepatocellular Carcinoma Metastasis via Enhancing Invasiveness and Angiogenesis,” Journal of Experimental & Clinical Cancer Research 39, no. 1 (2020): 20. 31973767 10.1186/s13046-020-1529-9 PMC6979009

[10] L. Fan , L. Yao , Z. Li , et al., “Exosome‐Based Mitochondrial Delivery of circRNA mSCAR Alleviates Sepsis by Orchestrating Macrophage Activation,” Advanced Science 10, no. 14 (2023): e2205692. 36965082 10.1002/advs.202205692 PMC10190648

[11] W. M. Pardridge , “Drug Transport Across the Blood‐Brain Barrier,” Journal of Cerebral Blood Flow and Metabolism 32, no. 11 (2012): 1959–1972. 22929442 10.1038/jcbfm.2012.126 PMC3494002

[12] X. Jiao , Y. Yu , J. Meng , et al., “Dual‐Targeting and Microenvironment‐Responsive Micelles as a Gene Delivery System to Improve the Sensitivity of Glioma to Radiotherapy,” Acta Pharmaceutica Sinica B 9, no. 2 (2019): 381–396. 30972284 10.1016/j.apsb.2018.12.001 PMC6437633

[13] X. Li , J. Tsibouklis , T. Weng , et al., “Nano Carriers for Drug Transport Across the Blood‐Brain Barrier,” Journal of Drug Targeting 25, no. 1 (2017): 17–28. 27126681 10.1080/1061186X.2016.1184272

[14] A. Vilella , G. Tosi , A. M. Grabrucker , et al., “Insight on the Fate of CNS‐Targeted Nanoparticles. Part I: Rab5‐Dependent Cell‐Specific Uptake and Distribution,” Journal of Controlled Release 174 (2014): 195–201. 24316476 10.1016/j.jconrel.2013.11.023

[15] J. Matsumoto , T. Stewart , W. A. Banks , and J. Zhang , “The Transport Mechanism of Extracellular Vesicles at the Blood‐Brain Barrier,” Current Pharmaceutical Design 23, no. 40 (2017): 6206–6214. 28914201 10.2174/1381612823666170913164738

[16] G. Morad , C. V. Carman , E. J. Hagedorn , et al., “Tumor‐Derived Extracellular Vesicles Breach the Intact Blood‐Brain Barrier via Transcytosis,” ACS Nano 13, no. 12 (2019): 13853–13865. 31479239 10.1021/acsnano.9b04397 PMC7169949

[17] Y. Zhang , Q. Liu , X. Zhang , et al., “Recent Advances in Exosome‐Mediated Nucleic Acid Delivery for Cancer Therapy,” Journal of Nanobiotechnology 20, no. 1 (2022): 279. 35701788 10.1186/s12951-022-01472-z PMC9194774

[18] J. Yang , X. Zhang , X. Chen , L. Wang , and G. Yang , “Exosome Mediated Delivery of miR‐124 Promotes Neurogenesis After Ischemia,” Molecular Therapy ‐ Nucleic Acids 7 (2017): 278–287. 28624203 10.1016/j.omtn.2017.04.010 PMC5415550

[19] M. Shao , S. Ye , Y. Chen , C. Yu , and W. Zhu , “Exosomes From Hypoxic ADSCs Ameliorate Neuronal Damage Post Spinal Cord Injury Through Circ‐Wdfy3 Delivery and Inhibition of Ferroptosis,” Neurochemistry International 177 (2024): 105759. 38735393 10.1016/j.neuint.2024.105759

[20] J. W. Yang , J. Ru , W. Ma , et al., “BDNF Promotes the Growth of Human Neurons Through Crosstalk With the Wnt/Beta‐Catenin Signaling Pathway via GSK‐3beta,” Neuropeptides 54 (2015): 35–46. 26311646 10.1016/j.npep.2015.08.005

[21] P. Thapak , G. Smith , Z. Ying , A. Paydar , N. Harris , and F. Gomez‐Pinilla , “The BDNF Mimetic R‐13 Attenuates TBI Pathogenesis Using TrkB‐Related Pathways and Bioenergetics,” Biochimica et Biophysica Acta ‐ Molecular Basis of Disease 1869, no. 7 (2023): 166781. 37286142 10.1016/j.bbadis.2023.166781 PMC10619508

[22] S. D. Chen , C. L. Wu , W. C. Hwang , and D. I. Yang , “More Insight Into BDNF Against Neurodegeneration: Anti‐Apoptosis, Anti‐Oxidation, and Suppression of Autophagy,” International Journal of Molecular Sciences 18, no. 3 (2017): 545. 28273832 10.3390/ijms18030545 PMC5372561

[23] Z. Li , F. Wu , D. Xu , Z. Zhi , and G. Xu , “Inhibition of TREM1 Reduces Inflammation and Oxidative Stress After Spinal Cord Injury (SCI) Associated With HO‐1 Expressions,” Biomedicine & Pharmacotherapy 109 (2019): 2014–2021. 30551457 10.1016/j.biopha.2018.08.159

[24] Y. Hao , R. Xiong , and X. Gong , “Memantine, NMDA Receptor Antagonist, Attenuates Ox‐LDL‐Induced Inflammation and Oxidative Stress via Activation of BDNF/TrkB Signaling Pathway in HUVECs,” Inflammation 44, no. 2 (2021): 659–670. 33174139 10.1007/s10753-020-01365-z

[25] C. E. McGregor and A. W. English , “The Role of BDNF in Peripheral Nerve Regeneration: Activity‐Dependent Treatments and Val66Met,” Frontiers in Cellular Neuroscience 12 (2018): 522. 30687012 10.3389/fncel.2018.00522 PMC6336700

[26] H. S. Shamsnia , A. Peyrovinasab , D. Amirlou , et al., “BDNF‐TrkB Signaling Pathway in Spinal Cord Injury: Insights and Implications,” Molecular Neurobiology 62 (2025): 1904–1944. 39046702 10.1007/s12035-024-04381-4

[27] C. L. Walker , X. Wu , N. K. Liu , and X. M. Xu , “Bisperoxovanadium Mediates Neuronal Protection Through Inhibition of PTEN and Activation of PI3K/AKT‐mTOR Signaling After Traumatic Spinal Injuries,” Journal of Neurotrauma 36, no. 18 (2019): 2676–2687. 30672370 10.1089/neu.2018.6294 PMC6727469

[28] H. Yin , L. Shen , C. Xu , and J. Liu , “Lentivirus‐Mediated Overexpression of miR‐29a Promotes Axonal Regeneration and Functional Recovery in Experimental Spinal Cord Injury via PI3K/Akt/mTOR Pathway,” Neurochemical Research 43, no. 11 (2018): 2038–2046. 30173324 10.1007/s11064-018-2625-5

[29] Z. Huang , J. Li , J. Wo , et al., “Intranasal Delivery of Brain‐Derived Neurotrophic Factor (BDNF)‐loaded Small Extracellular Vesicles for Treating Acute Spinal Cord Injury in Rats and Monkeys,” Journal of Extracellular Vesicles 14, no. 4 (2025): e70066. 40194993 10.1002/jev2.70066 PMC11975507

