# Supplementary material for: Exosome‐Loaded Engineered circBDNF Promotes Spinal Cord Injury Repair Through the PI3K/AKT/mTOR Signaling Axis
Source: CNS Neurosci Ther. 2026 Mar 19;32(3):e70784. doi: 10.1002/cns.70784 (PMC13093597; doi:10.1002/cns.70784)
Supplement: Supplementary file 1 — Table S1: Primers for qRT‐PCR analysis of gene expression. Table S2: Primary antibody information for immunofluorescence Table S3: Primary antibody information for Western blot. [file CNS-32-e70784-s001.docx]

**Supplementary Table 1. Primers for qRT-PCR analysis of gene expression.**

| Gene | Direction | Sequence (5'-3') |
| --- | --- | --- |
| CircBDNF | forward | AATACAGCAAAACTAGTGCCACC |
|  | reversed | CCCATTCACGCTCTCCAGAG |
| BDNF | forward | CGGTGTCGCCCTTAAAAAGC |
|  | reversed | CCGCTAGGAAGCCAACTTCA |
| GAPDH | forward | TACTAGCGGTTTTACGGGCG |
|  | reversed | TCGAACAGGAGGAGCAGAGAGCGA |

**Supplementary Table 2. Primary antibody information for immunofluorescence**

| Antibodies | Source | Identifier | Dilution |
| --- | --- | --- | --- |
| rat-anti-GFAP | abcam | ab279291 | 1:500 |
| rabbit-anti-NeuN | abcam | ab177487 | 1:100 |
| rabbit-anti-Iba1 | abcam | ab178846 | 1:2000 |
| rabbit anti-5-HT | immunostar | 20080 | 1:3000 |
| rabbit-anti-GAP43 | abcam | ab75810 | 1:3000 |
| Alexa Fluor 488 goat anti-rabbit IgG | abcam | ab150077 | 1:1000 |
| Alexa Fluor 594 goat anti-rabbit IgG | abcam | ab150080 | 1:1000 |
| Alexa Fluor 488 goat anti-rat IgG | abcam | ab150157 | 1:1000 |

**Supplementary Table 3. Primary antibody information for Western blot**

| Antibodies | Source | Identifier | Dilution |
| --- | --- | --- | --- |
| rabbit anti-BDNF | abcam | ab108319 | 1:1000 |
| rabbit anti-CD63 | Proteintech | 25682-1-AP | 1:1000 |
| rabbit anti-TSG101 | Proteintech | 28283-1-AP | 1:2000 |
| rabbit anti-Calnexin | Proteintech | 10427-2-AP | 1:5000 |
| rabbit anti-Bcl2 | Proteintech | 26593-1-AP | 1:1000 |
| rabbit anti-Caspase3 | Proteintech | 25128-1-AP | 1:1000 |
| rabbit anti-Caspase9 | Proteintech | 10380-1-AP | 1:800 |
| rabbit anti-TrkB | Proteintech | 13129-1-AP | 1:2000 |
| rabbit anti-Phospho-PI3K | Cell Signaling Technology | 17366T | 1:1000 |
| rabbit anti-Phospho-Akt | Proteintech | 80455-1-RR | 1:5000 |
| rabbit anti-Phospho-mTOR | Proteintech | 80596-1-RR | 1:5000 |
| rabbit-β-tubulin | Proteintech | 66240-1-Ig | 1:2000 |
